# Neuroendocrine Tumor of the Cauda Equina: A Report of a Rare Case With Histopathological and Immunohistochemical Correlation

**DOI:** 10.7759/cureus.103424

**Published:** 2026-02-11

**Authors:** Miguel Esquivel, Maria F Vargas Wille, Ariel Mendelewicz, Ana María Gutiérrez

**Affiliations:** 1 Neurosurgery, Hospital Mexico, San Jose, CRI; 2 Pathology, Universidad de Costa Rica, San Jose, CRI; 3 Neurosurgery, Universidad de Costa Rica, San Jose, CRI; 4 General Medicine, Universidad de Costa Rica, San Jose, CRI

**Keywords:** cauda equina, histopathology, immunohistochemistry, neuroendocrine tumor, rare case

## Abstract

Neuroendocrine tumors (NETs) of the cauda equina are rare, generally benign neoplasms. Previously known as paragangliomas, they were renamed as neuroendocrine tumors in the 2022 World Health Organization (WHO) classification of neuroendocrine neoplasms. These tumors typically occur in adults; however, cases have been reported in nearly all age groups.

This report describes the case of a 29-year-old male patient with chronic lumbar pain and bilateral radicular neuropathic pain. Magnetic resonance imaging (MRI) findings described an extramedullary intradural lesion at the level of L1-L2. Surgical resection was performed, resulting in rapid symptom resolution.

Clinical presentation and imaging findings are often nonspecific; therefore, definitive diagnosis relies on histopathological examination and immunohistochemical analysis. The main differential diagnoses include ependymoma, schwannoma, meningioma, and hemangioblastoma.

NET of the cauda equina is a rare entity, with only a few cases reported in the literature; therefore, this case report serves as a guide to establish a diagnosis and its possible surgical management for future patients.

## Introduction

Neuroendocrine tumors (NETs) in the cauda equina are very rare benign neoplasms, with approximately 300 cases reported in the literature since their first description in 1970 [1,2]. Previously, these tumors were misclassified as paragangliomas. Since the recent 2022 World Health Organization (WHO) classification of neuroendocrine neoplasms, they are now referred to as neuroendocrine tumors. This change in terminology is due to differences between the expression of various immunohistochemical markers and histological findings, as well as different clinical presentations. Spinal paraganglioma, most frequently found in the cauda equina, is a well-differentiated neuroendocrine tumor. In approximately 25% of cases, mature ganglion cells are present, hence the term “gangliocytic” tumors. Tumor cells typically express neuroendocrine markers such as chromogranin A and synaptophysin, with variable S100 immunoreactivity, while the sustentacular cells demonstrate intense nuclear and cytoplasmic S100 reactivity. These previously mentioned histological and immunophenotypic features initially supported classification as paragangliomas. However, subsequent studies showed that NETs lack the expression of GATA3, a transcription factor characteristic of paragangliomas, and instead express HOXB13, a transcription factor expressed in the spinal cord. Accordingly, the current WHO classification recognizes these lesions as cauda equina neuroendocrine tumors. Further studies are needed to better characterize their biomarker profile [3].

These tumors generally occur in adults, with a peak incidence between the fourth and sixth decade; nevertheless, cases have been reported in nearly all age groups, with a slight male predominance [1,2].

The main complaint among patients is low back pain and bilateral paresthesia in the lower extremities. Meanwhile, sphincter involvement with incontinence and erectile dysfunction is quite uncommon [4]. It is unusual to see cauda equina syndrome within the clinical presentation of these patients, even if spinal canal stenosis is significant; it may occur, but only in late stages [5,6]. These lesions are highly vascular and can bleed easily; sometimes, the manifestation may be that of a subarachnoid hemorrhage [5,7,8]. Despite being derived from neuroendocrine tissue, they usually appear to be non-functional [9,10].

For the study of NET of the cauda equina, magnetic resonance imaging (MRI) is the preferred imaging modality for diagnosis and follow-up. Clinical presentation and imaging findings can be very nonspecific for this entity [4,5]. Therefore, the definitive diagnosis is established through histopathological and immunohistochemical findings of the dissected lesion [11].

Other intradural extramedullary lesions with similar radiological characteristics are the main differential diagnosis, which are ependymoma, schwannoma, meningioma, and hemangioblastoma [5,9].

Case reports of NET of the cauda equina are scarce. There is little information on the clinical presentation, diagnosis, and management of this disease [12]. Hence, this case report presents valuable information for the medical community.

## Case presentation

A 29-year-old man, whose previous medical history was unremarkable, presented to the emergency department of Hospital México due to chronic lumbar pain and bilateral radicular neuropathic pain, ongoing for approximately one year. Sphincter continence was preserved, and there was no sexual dysfunction.

He had previously sought medical attention at another center, where a magnetic resonance imaging (MRI) documented a lumbar lesion that occupied 90% of the spinal canal. He was prescribed tramadol, paracetamol, deflazacort, and pregabalin for chronic pain management with an initial response, but the medication no longer provided pain relief. He was then referred to our medical center for further management of the MRI finding and possible surgical intervention.

At admission, his blood pressure was 160/93 mmHg, temperature 36.6°C, respiratory rate 16 breaths per minute, heart rate 87 beats per minute, and oxygen saturation 99% on ambient air. The patient had a body mass index (BMI) of 28.74. Regarding neurological examination, muscle strength was 5/5 and sensation was preserved. He showed bilateral knee hyperreflexia and normal ankle reflexes bilaterally, with a positive Lasegue sign on the right lower limb. The rest of the neurological and physical examination had no pathological findings.

**Figure 1:**
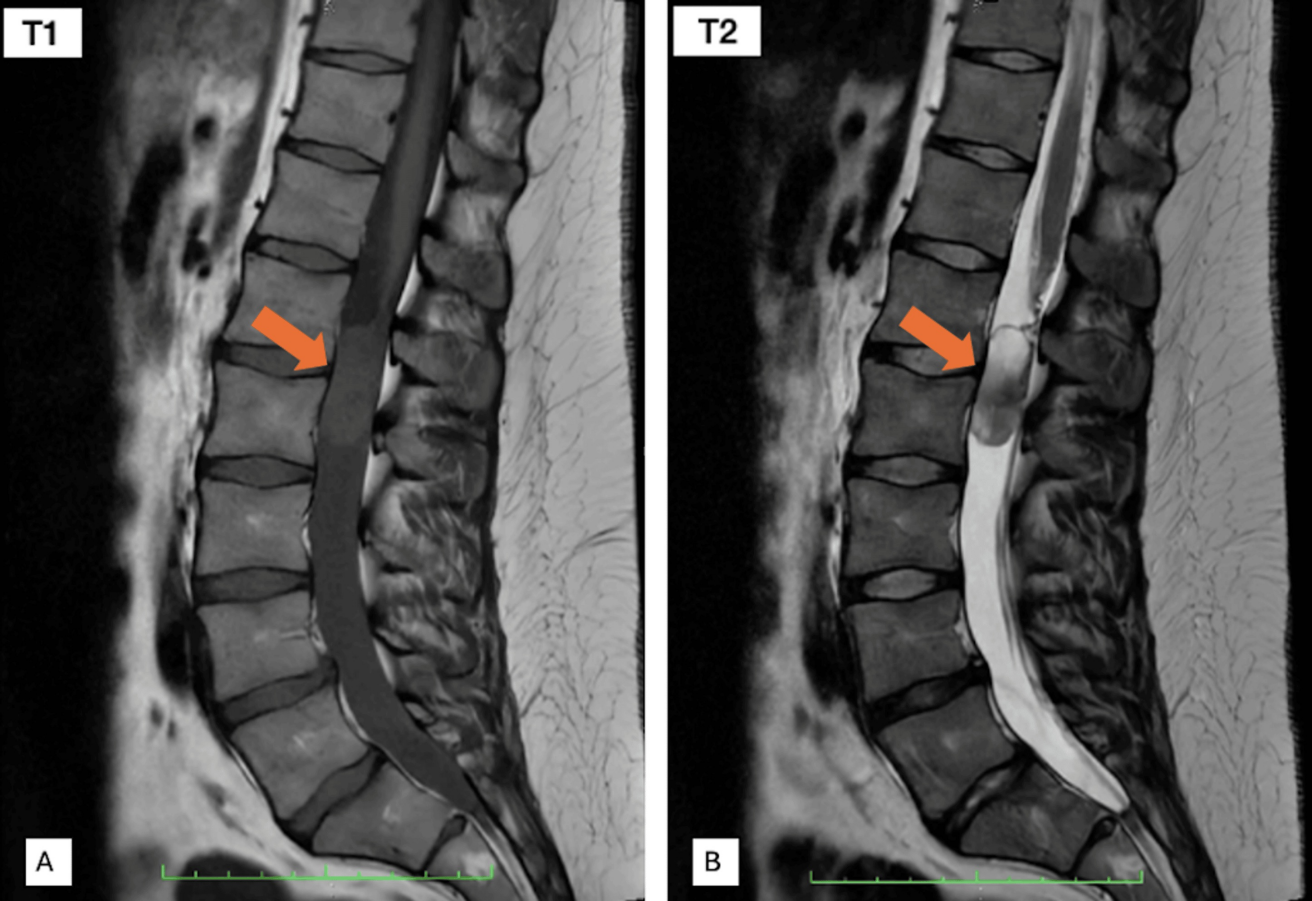
Sagittal MRI of the lumbar spine (A) T1-weighted sagittal image shows an intradural lesion (orange arrow) with an isointense signal relative to the spinal cord, with surrounding cerebrospinal fluid appearing hypointense. (B) T2-weighted sagittal image demonstrates the lesion (orange arrow) as hyperintense, with marked cerebrospinal fluid hyperintensity, resulting in clear delineation of the intradural component. MRI: magnetic resonance imaging

MRI documented an extramedullary intradural lesion of approximately 37×14 mm at the L1-L2 level, with isointensity in T1 and hyperintensity on T2 images (Figure 1). Laboratory data were retrieved retrospectively from available clinical records. Table 1 summarizes the laboratory parameters available at admission. Missing data reflects unavailability in the medical records.

**Table 1:** Available baseline laboratory values at hospital admission

Test	Result	Reference values
Hemoglobin (g/dL)	14	12.5-14.5
Hematocrit (%)	41.5	38-42
Leukocytes (×10^3^ mL)	11.7	4.5-10.0
Glycemia (mg/dL)	103	70-100
Creatinine (mg/dL)	0.94	0.6-1.2
Urea nitrogen (mg/dL)	17.7	7-25

**Figure 2:**
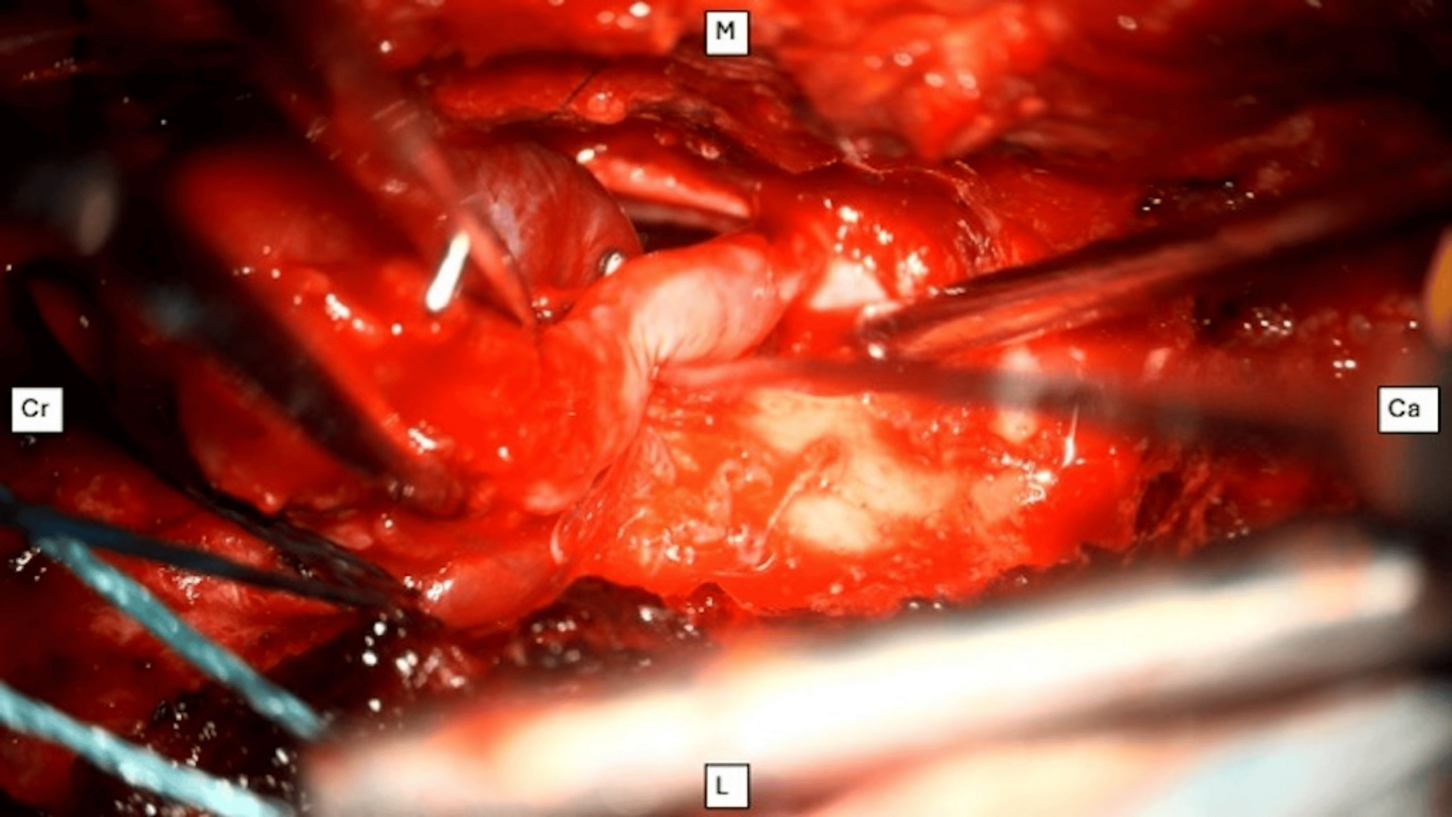
Intraoperative view of the cauda equina Resection of a neuroendocrine tumor, appearing as a well-defined, highly vascularized reddish mass. Anatomical orientation is indicated. Cr: cranial, Ca: caudal, M: medial, L: lateral

The patient underwent a laminectomy of right L1 and L2, durotomy, and macroscopic resection of the tumor with intraoperative microscopic evaluation. During surgery, the lesion appeared reddish and firm with elastic consistency and a tendency to bleed easily. A well-defined dissection plane was identified with no infiltration of the nerve roots, which were only contacted and displaced. Intraoperative pathology evaluation reported a round, blue cell tumor. Macroscopic complete resection of the tumor was performed (Figure 2).

**Figure 3:**
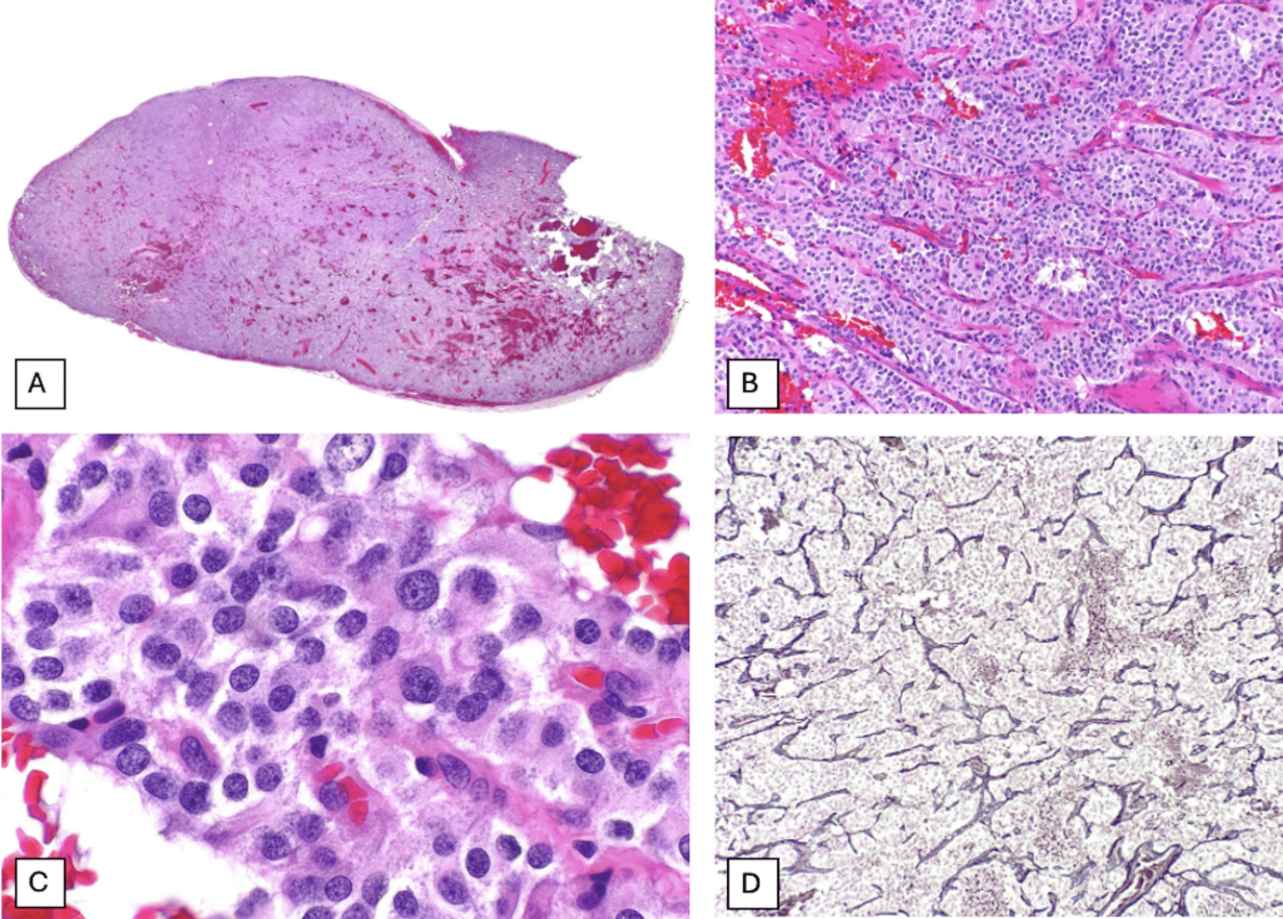
Histological features of the patient’s neuroendocrine tumor of the cauda equina (A) Low-power view showing a well-circumscribed lesion with prominent vascularization, readily appreciable even at this magnification. (B) The classic Zellballen pattern is observed, characterized by nests of tumor cells separated by a delicate capillary network. (C) At higher magnification, the tumor cells exhibit abundant eosinophilic cytoplasm, finely granular “salt-and-pepper” chromatin, and inconspicuous nucleoli. (D) Reticulin staining highlights the fine fibrous network surrounding the nests of chief (type I) cells.

Microscopically, the tumor was oval in shape, with a red-brown surface, measuring 2.2×1.1 cm. Histologically, it consisted of a well-circumscribed tumor with pushing borders, composed of large cells with abundant eosinophilic cytoplasm and round nuclei featuring fine salt-and-pepper chromatin and inconspicuous nucleoli, arranged in nests surrounded by a delicate capillary network. No mitotic activity was observed (Figure 3).

**Figure 4:**
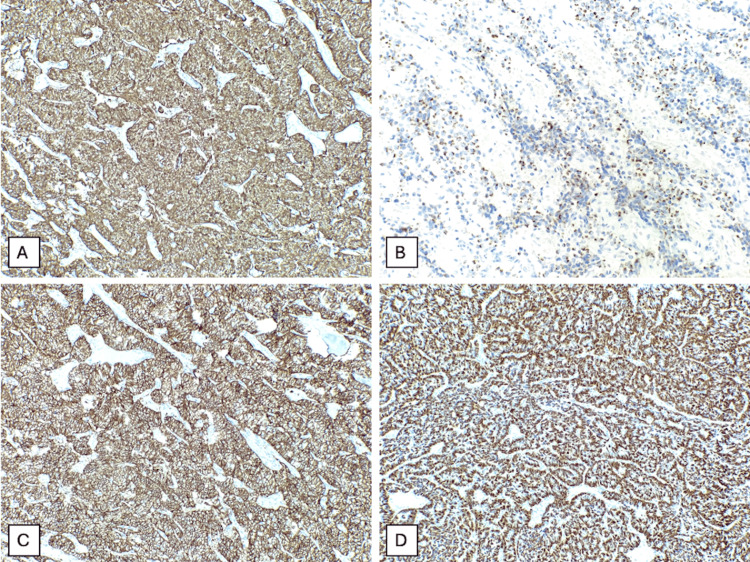
Immunohistochemical stains performed on the patient’s biopsy demonstrate diffuse synaptophysin positivity (A), heterogeneous chromogranin A expression (B), diffuse CD56 positivity (C), and cytoplasmic pancytokeratin staining in chief cells (D)

**Figure 5:**
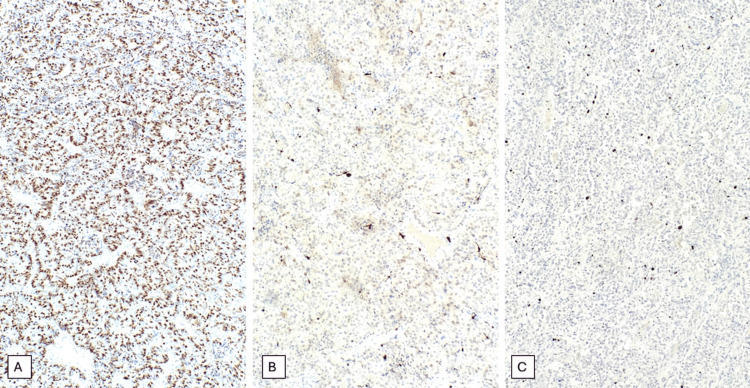
Immunohistochemical stains performed on the patient’s biopsy demonstrate diffuse cytoplasmic expression of CK CAM 5.2 (A), S100 shows focal positivity (B), and the Ki-67 proliferation index is approximately 2%-3% (C)

​​The neoplastic cells were immunoreactive for pancytokeratin (CAM 5.2), CD56, synaptophysin, and chromogranin, supporting the neuroendocrine nature of the lesion. S100 highlighted sustentacular cells surrounding the nests, and reticulin stain demonstrated the supporting fibrovascular network. The Ki-67 proliferation index was low, approximately 2%-3%. Immunohistochemical stains for CK7, CK20, EMA, and CD45 were negative. The morphological and immunohistochemical findings were consistent with the diagnosis of a neuroendocrine tumor of the cauda equina (Figure 4 and Figure 5).

After surgery, the symptoms resolved immediately. The patient was able to walk with no motor or sensory deficit, and no other neurological symptoms remained. The patient was discharged a few days later.

## Discussion

An extensive bibliographic review in medical databases such as PubMed, PubMed Central, and Science Direct revealed only a few case reports similar to our patient’s NET of the cauda equina. This entity represents a rare condition with limited cases reported in the literature. About 300-330 cases have been described since 1970, when the condition was first recognized [1,2].

The clinical presentation of our patient correlates with previously reported cases. The patient’s main symptom was lumbar pain, consistent with the findings described in the literature [6,13,14].

There are no pathognomonic imaging findings for the diagnosis of NET of the cauda equina. However, there are some typical features that can be found on MRI studies of these tumors, such as isointensity in T1, hyperintensity in T2, and enhancement with gadolinium administration [11,15].

Clinical features and MRI are not sufficient for establishing a definitive diagnosis. The confirmatory diagnosis relies on histopathological and immunohistochemical findings [11].

Histopathologically, NETs display the classical Zellballen architectural pattern, which translates to “ball of cells”, referring to nests or lobules of polygonal cells surrounded by sustentacular cells and fibrovascular stoma. These cells, also known as chief cells or type I cells, show a characteristic eosinophilic granular cytoplasm, central hyperchromatic nuclei with inconspicuous nucleoli, and “salt-and-pepper” chromatin, findings consistent with our patient’s biopsy [13,16,17].

With immunohistochemical stains, neuroendocrine markers such as synaptophysin, chromogranin A, INSM1, and CD56 are positive. Chief cells are also positive for vimentin and cytokeratins, with variable S100 immunoreactivity. The capillary and stromal network can be highlighted with reticulin staining, and it also contains S100-positive sustentacular or type II cells. The Ki-67 proliferation index role is not clear, although it may provide useful information; a low index supports a non-metastatic NET [3,16,17]. 

In the patient’s case and others reported in literature, the main differential diagnosis was ependymoma, due to similar MRI findings and location of the lesion (intradural extramedullary) [18]. The epithelial membrane antigen (EMA), which is frequently expressed in ependymomas, was negative in our case, making this diagnosis less likely [1]. Other ruled out less likely differential diagnoses were schwannoma, meningioma, and hemangioblastoma [11].

The prognosis is generally favorable for NET of the cauda equina after total surgical resection, with a low local recurrence rate [2]. However, recurrence can occur after subtotal removal. Although some authors suggest postoperative radiotherapy in cases of subtotal resection, its role in recurrence prevention has not conclusively been demonstrated [9].

Since there was no patient follow-up after discharge, we do not know how the patient’s condition progressed. This is considered to be a limitation of this case report and might be of interest for future investigations.

## Conclusions

Neuroendocrine tumors of the cauda equina are rare, generally benign neoplasms previously classified as paragangliomas. Due to nonspecific clinical and radiological features, definitive diagnosis relies on histopathological and immunohistochemical evaluation. Ependymoma remains the main differential diagnosis. Total surgical resection represents the treatment of choice and is associated with an excellent prognosis and low recurrence rates. Given the rarity of this entity, this case report contributes valuable information regarding diagnosis and surgical management for future patients.
